# Langerhans cell histiocytosis: a rare case of the multisystemic form in an infant^⋆^

**DOI:** 10.1016/j.abd.2021.06.011

**Published:** 2023-02-04

**Authors:** Thaís Oliveira Utiyama, Maria Laura Malzoni, Thalita Gabrieli Sanches Vasques, Cassiano Tamura Vieira Gomes

**Affiliations:** Faculty of Medical and Health Sciences of Sorocaba, Pontifícia Universidade Católica de São Paulo, Sorocaba, SP, Brazil

Dear Editor,

Langerhans cell histiocytosis (LCH) is an inflammatory neoplasm of myeloid precursor cells, in which there is an accumulation of specialized dendritic cells in different organs.^1^ This case report describes a rare case of LCH with a multisystemic presentation.

This case describes a two-month-old male patient, presenting erythematous-purpuric lesions scattered throughout the body since birth. After a short period of apparent improvement, the lesions recurred. There were no systemic manifestations.

On physical examination, angiomatous papules with hematic crusts on top and some flat, hypochromic, shiny papules were observed, also affecting the palmoplantar region and the oral cavity (Fig. 1).

**Figure 1:**
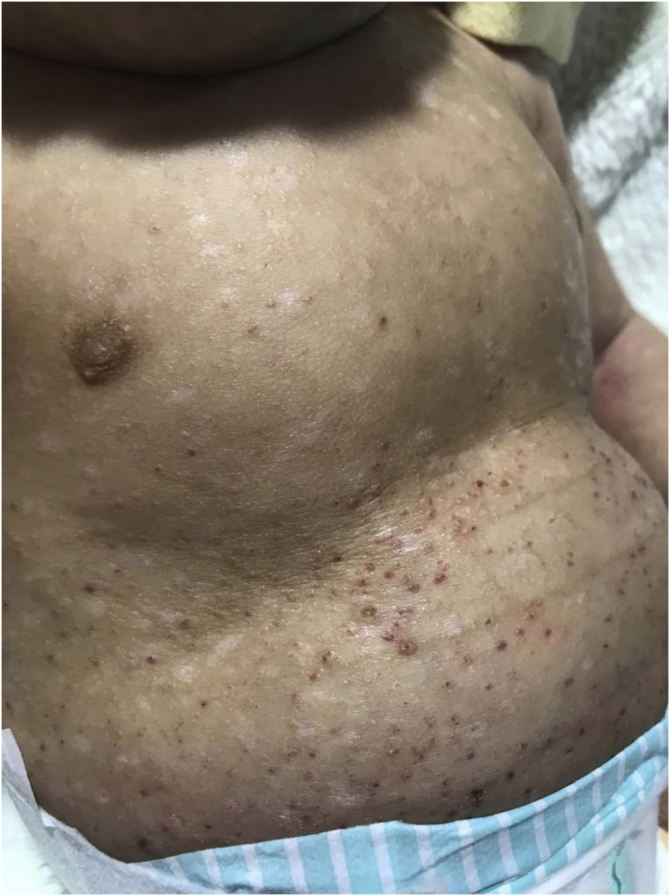
Angiomatous papules with hematic crusts on top and some flat, hypochromic papules, with a shiny appearance on the trunk.

Therefore, the diagnostic hypotheses of LCH, congenital cytomegalovirus (CMV), leukemia cutis, and severe combined immunodeficiency were suggested. A biopsy was performed, and serologies were requested (HIV, CMV, rubella, toxoplasmosis, VDRL) which were negative, whereas screening for congenital immunodeficiency showed no alterations.

Histopathology revealed chronic dermatitis associated with the presence of cells suggestive of Langerhans cells (Fig. 2). Immunohistochemistry showed positivity for CD1a, CD68, S100 protein; Ki67 was positive in 70% of the cells (Fig. 3). Once the diagnosis of LCH was confirmed, the investigation of other organs through a myelogram and computed tomography of the chest, abdomen, and pelvis led to the classification of the case as multisystemic LCH due to pulmonary and hepatic involvement. Treatment was started with weekly vinblastine 3 mg/m^2^ + prednisone 20 mg/m^2^, according to the *Brazilian Society of Histiocytosis* guideline. The condition improved significantly, but after about two months the skin lesions returned, and the pulmonary condition worsened, requiring oxygen therapy. A new chemotherapy regimen with Cladribine (2-CdA) was introduced, according to the Japanese protocol. The patient remains stable and is being followed by the oncology team.

**Figure 2:**
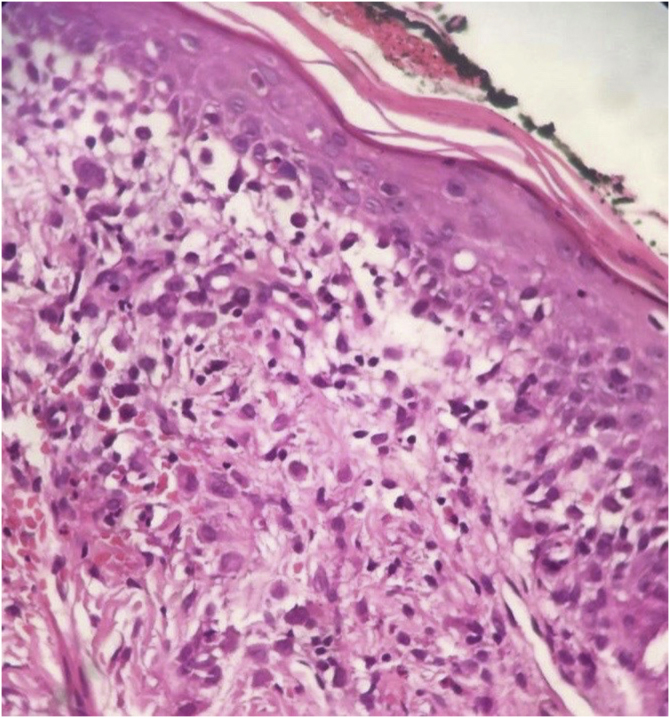
Chronic dermatitis associated with the presence of cells suggestive of Langerhans cells (Hematoxylin & eosin 100X).

**Figure 3:**
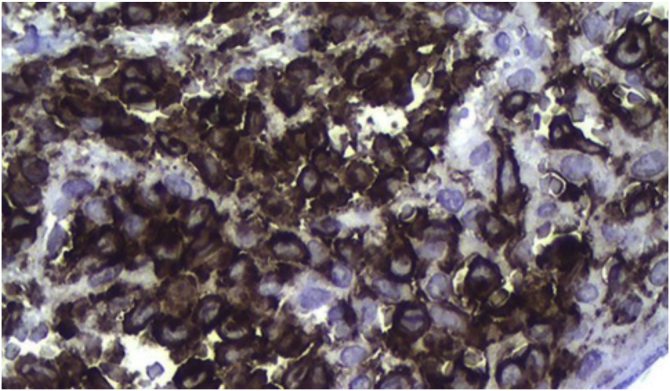
Positive immunohistochemistry for CD1a.

The incidence of LCH ranges from two to nine cases per million children under 15 years of age, with a peak between one and three years of age.^2^ It can affect one or multiple organs, with the following being considered at risk: liver, spleen, and bone marrow. Most patients have single-system involvement (70%).^1^

The most frequently affected organs are the bones, followed by the skin, but in infants, cutaneous manifestations are the main findings. Dermatologically, they present as a seborrheic-like dermatitis, and less often as hemorrhagic lesions, although these are favorable conducive to diagnosis.^3^

The pathogenesis remains unclear, with theories that support both a reactional and a neoplastic nature. Characterization as a neoplastic disorder is supported by the finding of a mutation in the BRAF V600E gene, and activation of the MAP kinase (MAPK) pathway.^4^

The diagnosis depends on the clinical, anatomopathological and immunohistochemical correlation. Histopathologically, varying amounts of Langerhans cells with a “coffee bean” appearance are observed. Positive immunohistochemistry for CD1a and CD207 (langerin) establishes the diagnosis.^5^

Treatment will depend on disease extent and severity. When the involvement is solely cutaneous, spontaneous resolution is common. For multisystem disease, treatment with systemic steroids and vinblastine for 12 months is the first-line regimen.^5^

LCH is a serious disease that, due to its very diverse clinical manifestation, is often diagnosed late, delaying treatment. The presence of a dermatologist in the clinical staff allows its early identification, with a significant impact on patient prognosis.

## Financial support

None declared.

## Authors' contributions

Thaís Oliveira Utiyama: Drafting and editing of the manuscript; critical review of the literature; critical review of the manuscript.

Maria Laura Malzoni: Effective participation in research orientation; intellectual participation in the propaedeutic and/or therapeutic conduct of the studied cases; critical review of the manuscript; approval of the final version of the manuscript.

Thalita Gabrieli Sanches Vasques: Critical review of the manuscript.

Cassiano Tamura Vieira Gomes: Approval of the final version of the manuscript.

## Conflicts of interest

None declared.
